# CTA Characteristics of the Circle of Willis and Intracranial Aneurysm in a Chinese Crowd with Family History of Stroke

**DOI:** 10.1155/2016/1743794

**Published:** 2016-01-04

**Authors:** Zhang-ning Jin, Wen-tao Dong, Xin-wang Cai, Zhen Zhang, Li-tong Zhang, Feng Gao, Xiao-kui Kang, Jia Li, Hai-ning Wang, Nan-nan Gao, Xian-jia Ning, Jun Tu, Feng-tan Li, Jing Zhang, Ying-jian Jiang, Nai-xin Li, Shu-yuan Yang, Jian-ning Zhang, Jing-hua Wang, Xin-yu Yang

**Affiliations:** ^1^Department of Neurosurgery, Tianjin Medical University General Hospital, Tianjin Neurological Institute (TNI), 154 Anshan Road, Heping District, Tianjin 300052, China; ^2^Department of Neuro-Epidemiology, Tianjin Medical University General Hospital, TNI, 154 Anshan Road, Heping District, Tianjin 300052, China; ^3^Department of Radiology, Tianjin Medical University General Hospital, 154 Anshan Road, Heping District, Tianjin 300052, China

## Abstract

*Background and Purpose.* The vascular morphology in crowd with family history of stroke remains unclear. The present study clarified the characteristics of the intracranial vascular CoW and prevalence of intracranial aneurysms in subjects with family history of stroke.* Methods.* A stratified cluster, random sampling method was used for subjects with family history of stroke among rural residents in Jixian, Tianjin, China. All the subjects underwent a physical examination, head computed tomography (CT) scan, and cephalic and cervical computed tomography angiography (CTA) scan. Anatomic variations in the Circle of Willis and cerebrovascular disease in this population were analyzed.* Results.* In the crowd with similar living environment, stable genetic background, and family history of stroke and without obvious nerve function impairment (1) hypoplasia or absence of A1 segment was significantly different in gender (male versus female: 9.8% versus 18.8%, *p* = 0.031), especially the right-side A1 (male versus female: 5.9% versus 16.4%, *p* = 0.004). (2) Hypoplasia or absence of bilateral posterior communicating arteries was more common in men than women (58.2% versus 45.3%, *p* = 0.032). Unilateral fetal posterior cerebral artery was observed more often in women than men (17.2% versus 8.5%, *p* = 0.028). (3) The percentage of subjects with incomplete CoW did not increase significantly with age. Compared to healthy Chinese people, the crowd had a higher percentage of incomplete CoW (*p* < 0.001). (4) No obvious correlation between risk factors and CoW was found. (5) The prevalence of aneurysm was 10.3% in the special crowd.* Conclusions.* The certain variations of CoW showed significant relation to gender, but not to age in people with family history of stroke. The incomplete circle may be a dangerous factor that is independent of common risk factors for stroke and tend to lead to cerebral ischemia in the crowd with family history of stroke. The prevalence of intracranial aneurysm is comparatively high in the present subjects compared to other people.

## 1. Introduction

In China, stroke has exceeded heart disease to become the leading cause of death and adult disability, which causes approximately 116 deaths in population of 100 000 in urban areas and 111 deaths in rural areas [1]. Strokes are usually subdivided into ischemic stroke and hemorrhagic stroke; ischemic stroke is responsible for 87% of stroke [2]. There are various recognized risk factors for ischemic stroke which include age, race, family history, hypertension, atherosclerosis, diabetes, cardiac arrhythmias, hyperlipidemia, and cigarette smoking. Gene-environment interactions are important reasons for the pathogenesis of stroke. Moreover, it is widely acknowledged that stroke has an important hereditary component, and several studies also indicated the association between family history of stroke and stroke risk [3, 4]. However, all the studies in population with family history of stroke did not explore the relevant cerebrovascular morphology, especially the morphology of Circle of Willis (CoW), which is directly related to cerebral blood supply.

Approximately 400 years ago, Thomas Willis had described the arterial circle located at the bottom of the cerebrum. It connects the left internal carotid artery (ICA), right ICA, and vertebrobasilar artery via communicating arteries. Morphological analysis showed that approximately 42% of the general population has complete CoW [5–8]; thus, anatomical variations exist in over 50% of the population. Variations in the CoW are often caused by changes in the diameter of the vessels, including hypoplasia, agenesis, and the addition of certain branches [9]. Cow has two characteristics; one is compensatory ability, which is related to the presence of arteries and their diameter [10]. Some patients with CoW variations have sufficient compensatory ability; the risks of developing transient ischemic attack (TIA) and stroke are relatively low in such subjects [11, 12]. Patients with severe carotid artery stenosis or occlusion can redistribute blood to ischemic areas to improve cerebral perfusion via the CoW, which can prevent ischemic events and their progression [13–15]. Another characteristic is that CoW variations are also related to the occurrence of aneurysms.

Our previous epidemiological survey found significant phenomenons for incidence rate of stroke among the population in some rural areas of Jixian, Tianjin, China [16, 17], which we named it as “J group.” The “J” group has several characteristics such as (1) 95% of the village populations are low-income farmers; (2) most are ethnic Han according to the household registration system and traditional customs, living in the same or nearby villages for several generations; (3) annual immigration was 0.23% from 1992 to 2012; (4) the living environment is stable, with carbohydrates (mainly wheat flour) and pork as staples; all the above constituted both stable genetic background and acquired living environment for these persons and make those impacts on the variation of CoW as less as possible. In the present study, the main objective is to clarify the morphological characteristics of CoW and investigate the prevalence of intracranial aneurysms in subjects with a stroke family history from “J group.”

## 2. Materials and Methods 

### 2.1. Study Population

The Ethics Committee of Tianjin Medical University approved this study. Informed consent was obtained from all subjects. The used dates were from a survey that investigated secular trends of first-ever stroke in rural northern China over 2 decades. The study population is within 18 administrative villages. Because MRI became available in 2006 and the proportion of imaging diagnosis (CT/MRI) improved dramatically in recent decades, we mainly adopt the date of 2006–2012 period. During that period, we identified 471 patients with a first-ever stroke in these villages.

This research takes these 471 persons as probands. First, these villages were divided into three strata by geographical location: east, south, and north. This research randomly sampled one village from each stratum. There are 75 probands in the 3 villages. The research regards their immediate family members as a crowd with family history of stroke, which contained 340 individuals alive. The subjects involved in the present study were selected from these immediate families that did not contain these probands, because the probands were diagnosed with fully clinical strokes, with evident neurological dysfunction, which may be attributed to severely damaged vessels (e.g., vessel occlusion) and interfere with the analysis of CoW. The selected persons should meet the inclusion criteria: (1) Permanent residents ≥ 18 years without a history of immigration or emigration; (2) no obvious neurologic impairment (modified Rankin Scale [mRS] ≤ level 1); (3) subjects with a history of acute myocardial infarction or a mental disorder preventing them from independently answering questions were excluded. 286 residents (84.1%) met the criterions and underwent all required questionnaire and physical and image examinations at the General Hospital of Tianjin Medical University. The final number of residents whose date was analyzed included in this study was 281 (153 men, 54.4%; 128 women, 45.6%), after excluding 3 individuals for whom physical examination data were incomplete and one individual with a history of hyperthyroidism and another one with an allergic reaction to the contrast agent.

### 2.2. Questionnaire and Physical Examination

A questionnaire was designed by the Department of Epidemiology of the General Hospital of Tianjin Medical University for face-to-face interviews and was used to collect the following information: demographic data, including name, gender, date of birth, and educational level; previous history of hypertension, diabetes, polycystic kidney disease, atrial fibrillation, arrhythmia, stroke, and TIA (stroke was considered present if objective imaging evidence could be provided; TIA was considered present when clinical symptoms of neurological dysfunction occurred, but no objective evidence suggested brain stroke); and medication history. Participants were deemed to be smokers if they consumed >100 cigarettes in the past year. Alcohol consumption was defined as >30 g alcohol per week for at least one year. The physical examination was as follows: blood pressure (BP), body height, weight, and BMI (BMI was calculated as weight in kg divided by height in m squared. Subjects with a BMI from 25.0 to 29.9 and ≥30 kg/m^2^ were classified as overweight and obese, resp.). Subjects also underwent hematological examinations. Hypertriglyceridemia was defined as total triglycerides > 150 mg/dL; low HDL-C was defined as <40 mg/dL in men and <50 mg/dL in women [18].

### 2.3. CT and CTA

Scanning was performed on a 64-section cardiac CT scanner (CT750 HD, GE, America) with a standardized optimized contrast-enhanced protocol (120 kV [peak]; 180 mAs; collimation, 64 × 0.625 mm; rotation time, 0.5 s; pitch, 1.375). The head CT scanning range was from the carotid bifurcation to the parietal. All subjects were injected with 60 mL contrast agent (iopromide 370 mg/mL, Bayer) followed by a 40 mL saline bolus chaser both at an injection rate of 4 mL/s. Image reconstructions were made with a field of vision of 250 mm, matrix size of 512 × 512, and section thickness of 0.625 mm. The scan delay time was determined by a bolus-tracking technique with a region of interest at one internal carotid artery. When a threshold of 50 HU was exceeded, the spiral scan was automatically started; then, coronal and sagittal multiplanar reformats as well as maximum intensity projections (MIP) and 3D volume-rendered (VR) images were created at a GE Advantage Workstation.

### 2.4. Image Analysis

CoW configuration was evaluated and measured on the basis of source images as well as 3D VR and MIP angiogram. First, a chief neurosurgeon (>20 years of experience in interpreting CTA images) and a neuroradiologist (>15 years of work experience, the person who performed CTA) interpreted the CTA images independently and recorded the results. Other neuroradiology experts were consulted if the two persons' opinions differed; then they would discuss together, until they arrive at a result.

The vessels evaluated including bilateral precommunicating anterior cerebral artery (A1 segment), anterior communicating artery (AcomA), posterior communicating artery (PcomA), and bilateral precommunicating posterior cerebral artery (P1 segment). A vessel diameter of 1 mm was classified as normal. Arterial segments < 1 mm were considered hypoplastic. Undeveloped segments on the CTA were considered absent.

**Figure 1:**
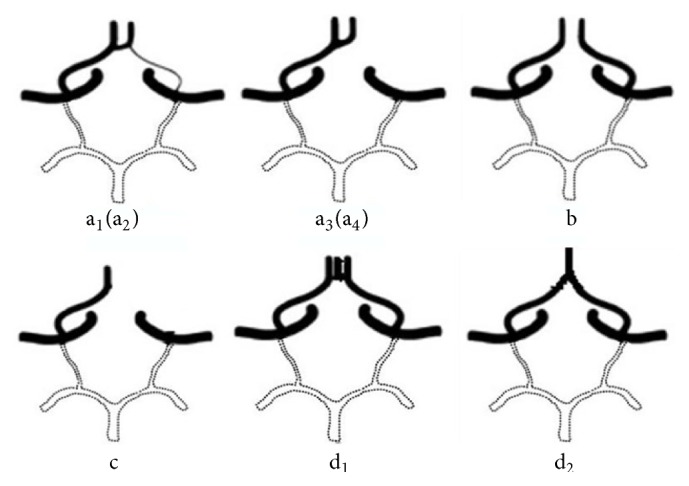
Anterior CoW variants a_1_. Hypoplastic left-side A1 segment a_2_. Hypoplastic right-side A1 segment a_3_. Absence of left-side A1 segment a_4_. Absence of right-side A1 segment b. Absence or hypoplasia of the AcomA c. Hypoplasia of unilateral anterior cerebral artery d_1_. Trifurcation of the anterior cerebral artery d_2_. The azygos anterior cerebral artery.

**Figure 2:**
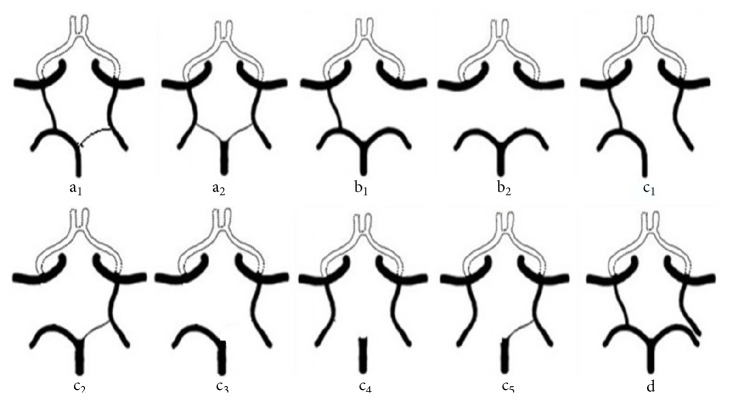
Posterior CoW variants a_1_. Unilateral FTP (P1 ≥ 1 mm) and contralateral PcomA patent a_2_. Bilateral FTP (bilateral P1 ≥ 1 mm) b_1_. Absence or hypoplasia of unilateral PcomA b_2_. Hypoplasia or absence of both PcomA c_1_. Unilateral FTP and hypoplasia or absence of the ipsilateral P1 c_2_. Unilateral FTP (ipsilateral P1 ≥ 1 mm) and hypoplasia or absence of the contralateral PcomA c_3_. Unilateral FTP (hypoplasia or absence of the ipsilateral P1) and hypoplasia or absence of the contralateral PcomA c_4_. Bilateral FTP with hypoplasia or absence of both P1 segments c_5_. Bilateral FTP with hypoplasia or absence of one P1 segment d. Unilateral FTP and ipsilateral complete posterior cerebral artery; they do not have direct contact.

The classification of the anterior and posterior CoW circulation parts was mainly based on angiodysplasia and agenesis (Figures 1 and 2). Anterior CoW variant types a_1_–c_2_ and d_1_ and d_2_ were classified as incomplete and complete, respectively. There were three configurations of the posterior CoW according to the relationship between the P1 segment and ipsilateral PcomA: fetal, transitional, and adult [19]. In the fetal configuration, the diameter of the P1 segment is absent or less than that of the ipsilateral PcomA. Thus, the blood supply to the occipital lobes is primarily via the ICA. Posterior CoW with fetal-type posterior cerebral artery (FTP) also was eligible for classification as complete, provided that all components of the posterior circle were ≥1 mm, including both P1 segments. Posterior variant types a_1_ and a_2_ are complete, whereas the remainders are incomplete.

The gross CoW morphology was assessed according to the completeness of the anterior and posterior parts. The overall morphology of the CoW was classified as entirely complete (EC), partially complete (PC), or incomplete (IC). The CoW was classified as EC if all component vessel segments of both anterior and posterior parts were visible, intact, and ≥1 mm in diameter. PC referred to CoWs with a complete and continuous anterior or posterior configuration with vessel diameters of ≥1 mm. Thus PC can be divided into 2 types, which were incomplete anterior type (A-PC) and incomplete posterior type (P-PC). IC was defined as when both the anterior and posterior parts had a defective vessel segment.

In the imaging studies, aneurysms ≥ 2 mm in diameter or diagnosed previously were included in the analysis [20, 21]. A five-point standardized scale from zero to four was used in the diagnosis of the aneurysms [20], with zero indicating absent and four as definitely present; an aneurysm was considered positive when the score was at least three.

### 2.5. Analysis and Statistics

The present data were screened and compared against those of 160 Chinese individuals with unknown family history of stroke that was from the study of Li et al. [7]. All data were analyzed using SPSS version 19.0. The differences were analyzed by independent sample* t-*tests or the *χ* ^2^ test. Fisher's exact test was used in cases with a theoretical frequency less than five. The associations of aneurysm prevalence and age-related changes were analyzed by linear-by-linear association using the *χ* ^2^ test. All *p* values were two-sided, and a *p* value < 0.05 was considered statistically significant.

## 3. Results

### 3.1. The Results of Questionnaire and Physical Examination

281 subjects (153 men, 54.4%; 128 women, 45.6%) aged 24–77 years (mean: 50.9 ± 10.5 years) were analyzed; there was no difference in age between sexes (*p* = 0.887). A history of cerebral infarction diagnosis by secondary or higher level healthcare institutions was found in 17 of them. Two subjects had a history of spontaneous subarachnoid hemorrhage (SAH): one had undergone AcomA aneurysm embolization treatment, and the other one was SAH of unknown origin after examination (they could provide information from the time of SAH). Therefore, 19 subjects (ten men, nine women) aged 36–77 years (mean: 59.7 ± 9.2 years) had a history of cerebral stroke. Subjects with hypertension were most common (56.6%) among the risk factors. The percentage of hypertriglyceridemia was significantly higher in women (*p* < 0.001). However, the proportion of subjects who smoked was more common in men (*p* < 0.001). 8.9% of the subjects were diabetics. The other basic characteristics of all subjects are shown in Table 1.

### 3.2. Preliminary Imaging Examination Results

Head CT revealed brain infarctions of various degrees and different areas in 36 subjects; 19 of them were first observed by the CT examination. Therefore, there were a total of 36 (12.8%) ischemic strokes and 38 (13.5%) strokes in the selected crowd. The head CT of one SAH patient who underwent intracranial aneurysm embolization showed a dense shadow of a metal object. CT also revealed intracranial arachnoid cysts in three subjects (located on the left temporal pole, left parietal-occipital, and right temporal region, resp.).

CTA showed intracranial aneurysms in 29 subjects (10.3%, including patients having undergone embolization) including one with two aneurysms in the two C7 segments (according to the Bouthillier segmentation method) of the left and right carotid arteries. Therefore, there were a total of 30 intracranial aneurysms.

### 3.3. Anterior CoW Variants

A total of 6 variations in the anterior CoW are shown in Table 2. Anatomic variations were present in 125 subjects (44.5%), 65 of whom were women (50.8%) and 60 were men (39.2%); there was no significant difference between sexes (50.8% versus 39.2%, *p* = 0.052). There were 121 subjects with variations involving an incomplete anterior CoW, including 58 men (37.9%) and 63 women (49.2%), respectively (37.9% versus 49.2%, *p* = 0.057). Agenesis of the AcomA was most prevalent (83, 29.5%), including 44 men (28.8%) and 39 women (30.5%) (28.8% versus 30.5%, *p* = 0.75). A1 segment variations (a_1_, a_2_, a_3_, a_4_, and c in Figure 1) were found in 39 subjects (13.9%) including 15 men (9.8%) and 24 women (18.8%) (9.8% versus 18.8%, *p* = 0.031). Right-side A1 variations were found in 30 subjects (10.7%) including 9 men (5.9%) and 21 women (16.4%) (5.9% versus 16.4%, *p* = 0.004).

### 3.4. Posterior CoW Variants

There were 10 types of variations in the posterior CoW (Table 3). There were 226 subjects (80.4%) with one of these types of variants, including 124 men (81.0%) and 102 women (79.7%) (81.0% versus 79.7%, *p* = 0.78). The prevalence of incomplete variants was significantly higher in posterior circle than anterior circle (75.8% versus 43.1%, *p* < 0.001). Hypoplasia or absence of both PcomA was most prevalent (147 subjects, 52.3%), including 89 men (58.2%) and 58 women (45.3%) (58.2% versus 45.3%, *p* = 0.032). There were 21 men (13.7%) and 26 women (20.3%) when FTP variants were included (13.7% versus 20.3%, *p* = 0.14). Unilateral FTP (35 subjects, 12.5%; 13 men, 8.5%; 22 women, 17.2%; *p* = 0.028) was observed more often in women. Types c_2_ and c_3_ were observed most often (20 subjects, 57.1%) among the unilateral FTP variations. There were 12 subjects with bilateral FTP (4.3%; eight men, four women).

### 3.5. Combined Analysis of the Entire CoW

Table 4 shows the relationships of entire CoW with sex and age. There were no significant differences in the gender or age specific prevalence of the entire circle morphology. Only 43 subjects (15.3%) possessed an entirely complete circle. In all age groups, the morphology of P-PC possesses the largest proportion. An entirely complete circle was found slightly higher in men than women (17% versus 13.3%, *p* = 0.39). The rate of A-PC form decreased with age, but not significantly (*p* = 0.36). The percentage of incomplete CoW was slightly higher in women than men (*p* = 0.32).

### 3.6. Comparison with Generally Healthy Chinese Subjects (Table 5)

This study used the inclusion criteria from a previous study to select subjects for comparison with a healthy Chinese population in cerebrovascular diseases [7]. The inclusion criteria were as follows: (1) no history of transient cerebral ischemic attacks, acute ischemic stroke, or hemorrhagic stroke; (2) no disabling neurological impairment found in examinations; and (3) no brain abnormalities. Overall, 216 subjects (Table 5; 118 men, 98 women) aged 24–70 years (mean: 49.7 ± 10.1 years) met the criteria; there were no statistically significant sex-related differences in the age (*p* = 0.68). The control group consisted of 160 subjects from Li et al. (82 men, 78 women; mean age: 46 years); this group had a significantly lower percentage of entirely complete CoW (14% versus 27%, *p* = 0.003) and a higher percentage of incomplete CoW (36% versus 17%, *p* < 0.001).

### 3.7. The Correlation of Risk Factors and the Circle of Willis

Table 6 shows the different prevalence of the entire circle morphology for subjects with different risk factors. The prevalence of P-PC was significantly higher in subjects without hypertension than those with hypertension (49.2% versus 35.8, *p* = 0.025). However, there were no significant differences in the other risk factors.

### 3.8. Intracranial Aneurysms

#### 3.8.1. Age and Sex Specific Prevalence of Intracranial Aneurysms

The relationship between aneurysms and sex and age is shown in Table 7. The prevalence rate of aneurysm was 10.3% (95% CI: 6.7–13.9%). Aneurysm patients were 28–70 years old (mean: 49.4 ± 10.7 years); there were 15 men (9.8%, 95% CI: 5.0–14.6%; mean age: 48.7 ± 11.8 years) and 14 women (10.9%, 95% CI: 5.5–16.4%; mean age: 50.1 ± 9.6 years). There was no significant difference in the prevalence rate of aneurysms between sexes (*p* = 0.76). There was also no significant difference between the ages of male and female aneurysm patients (*p* = 0.72). As shown in Table 7, the overall aneurysm prevalence rate and that in men decreased with increasing age but not significantly. Out of the 280 patients without a history of aneurysms or aneurysmal SAH, 28 patients had unruptured cerebral aneurysms (UCAs) (10.0%, 95% CI: 6.5–13.5%), including 15 men (9.8%, 95% CI: 5.0–14.6%) and 13 women (10.2%, 4.9–15.6%) (*p* = 0.90). The mean ages of the UCAs group and the group without aneurysms/aneurysmal SAH were 48.9 ± 10.6 and 51.0 ± 10.4 years, respectively (*p* = 0.32).

#### 3.8.2. Location of Intracranial Aneurysms

There were 19 (63.3%) aneurysms at various locations on the internal carotid artery, including nine (47.4%) on the C5-6 segments and 7 (36.8%) in the C7 segment (according to the Bouthillier segmentation method). Three aneurysms (10%) were located on the vertebrobasilar artery, two (6.7%) on the A2 segment, and three (10%) on the anterior communicating artery. There was one aneurysm each on the middle cerebral, posterior cerebral, and posterior inferior cerebellar artery. There were six fusiform aneurysms: three on the posterior circle, one on the M1 segment of the middle cerebral artery, one on the internal carotid artery, and one on the cerebral anterior A2 segment.

## 4. Discussion

Early research on the CoW was primarily based on autopsy data [5, 22]. Limitations may lie in the arteries losing their true form due to formalin immersion. Head CTA is a fast, less expensive, and noninvasive method of intracranial vessel examination. Its high sensitivity (81–90%), specificity (93%) [20], and accuracy in the detection of intracranial aneurysms as well as the anatomic variations, stenosis, and occlusion of intracranial vessels exceed those of conventional MRA [8, 23, 24]. The present study used the latest CTA technology to investigate intracranial arterial morphology in subjects with family history of stroke for the first time. The selected crowd also has the several characteristics: (1) the persons have similar acquired living environment and relatively stable genetic background on account of several generations living in the same or nearby villages; (2) the crowd have no obvious neurological impairment.

## 5. The Circle of Willis

The percentage of complete anterior CoWs ranges from 74 to 90% in different groups [7, 25–27]. The present study showed that only 56.9% of subjects with family history of stroke had a complete anterior CoW. The absence or hypogenesis of the AcomA was the most common CoW variant (83 subjects, 29.5%); this proportion is similar to the autopsy results of Fisher (29.0%) and Eftekhar et al. (29.5%) mentioned in previous literature [26], but higher than that in imaging studies, and the prevalence is only 9.4% in the head CTA of normal Chinese subjects [7]. Furthermore, the prevalence of A1 variations (particular the right-side A1 variations) was significantly higher in women. Hypogenesis or agenesis of the A1 segment is related to symptoms caused by carotid artery stenosis [28]. In addition, hypogenesis or absence of either AcomA or A1 segment could cause evident left-side hypoperfusion during unilateral selective cerebral perfusion [29]. Analysis of anterior circulation showed that when stenosis or occlusion was induced in a carotid artery on one side, symptoms of cerebral ischemia may be more likely to be present in this population. Particularly when pathological changes occurred in the left carotid artery, more women would suffer from the symptoms caused by left cerebral hemisphere ischemia. This may play a role of prediction in the development of ischemic stroke.

In previous imaging research [7, 25, 27, 30], the percentage of complete posterior CoW ranges from 31 to 52% compared to 18.1% in the present study. The percentage of incomplete variations in the posterior CoW was statistically higher than that of the anterior CoW. Agenesis or hypoplasia of the bilateral PcomA was the most common variation of the posterior circulation; the percentage is significantly higher in men than women. The CoW can quickly respond to cerebral hypoperfusion by simply changing the direction of blood flow, and the communicating artery is the most efficient channel for compensation in the CoW [31–33]. When the left posterior or bilateral posterior communicating artery in the CoW is incompletely developed, unilateral selective cerebral perfusion is most likely to cause the occurrence of stroke in the left side of the brain [34].

The other commonly observed variation in the posterior CoW was FTP. In imaging studies [7, 25, 35–37], the prevalence rate of FTP ranges from 3 to 32% compared to 16.4% in the present study; this large discrepancy in prevalence may be related to the study populations, classification, and selection standards. A study has pointed out that unilateral FTP is significantly more common in women [38]. In the present crowd, unilateral FTP was also significantly more often in women than men. The other studies do not report this phenomenon. FTP is one of the major variations in the posterior CoW that makes the carotid artery the only blood supply to the middle and posterior cerebral arteries, resulting in poor leptomeningeal collaterals between the carotid artery and vertebrobasilar artery system. Hence, this becomes a risk factor for stroke [39]. FTP, which is considered a variation of ipsilateral PcomA absence, is also related to border zone infarction [32, 39].

According to the above results, subjects with family history of stroke, especially men, have a greater risk for the symptoms of ischemic cerebrovascular disease in the short term when the basilar artery suffers from stenosis lesions. However, more women would have ischemic symptoms caused by posterior circulation ischemia due to the poor leptomeningeal collaterals with the continuous extension and development of lesions.

Recent imaging research shows that the proportion of EC CoW ranges from 27 to 89.7% in various groups [7, 25, 40, 41]. The prevalence of EC morphology was only 15.3% in the crowd with family history of stroke, and compared to the general population in China, the present subjects had significantly greater prevalence of incomplete CoW. The compensatory ability of CoW is related to cerebral ischemic incidents [12]. The present crowd with family history of stroke may be more vulnerable to ischemia. Consistent with previous study, no statistically significant age- or sex-related differences of the entire circle morphology in population with family history of stroke were found. Thus, anatomic variations of the CoW may be genetically determined [22, 42]. The PC form was divided into A-PC and P-PC morphology in the present study. The dates showed that P-PC form was most common in all age groups, which was likely due to the higher prevalence of incomplete posterior circle. A decreased tendency was found in A-PC form; the slightly decreased cause may be due to sampling. The dates also showed that incomplete circle was slightly high in women. Previous study has found that the diameter has a general trend of less mean diameters in the elderly and women [25]. These may be a reminder that more attention may be emphasized in women in later life.

The prevalence of stroke was 2.5% among Whites and 2.4% among Asians [43]. The age adjusted stroke prevalence in China varies from 259.86 to 719 per 100 000 for all ages [44]. However, there was higher prevalence (13.5%) in the present crowd. The percentages of hypertension and diabetes were 51.7% and 3.7%, respectively, in Ji Country, and the average of BMI was 25.23 in an epidemiological investigation [45]. However, the related prevalence and average were higher in the present crowd with family history of stroke compared to the overall level of the population in Jixian. These recognized risk factors contribute to the development of stroke. Mentioned in the previous content, incomplete CoW would be a risk factor for stroke in the present crowd as well. We studied the relation between risk factors and the morphology of CoW. However, no obvious correlation was found, except significant lower prevalence of P-PC in subjects with hypertension (the specific reason remains unclear). It is possible that these recognized risk factors affect the development of stroke by damaging the large and small vessels primarily rather than medium-sized vessels of CoW directly in crowd with family history of stroke. Hence, the incomplete CoW may be a risk factor that was not related to the common risk factors for ischemic stroke, which make the crowd tend to suffer from cerebral ischemia.

### 5.1. Aneurysms

The prevalence rate of aneurysms discovered through autopsy is 4.6%; furthermore, the prevalence rate in women is 2.4 times that in men, and many aneurysms are located on the middle cerebral artery [46]. Imaging studies show that the prevalence rate of UCAs in subjects with no medical history of aneurysm or aneurysmal SAH ranges from 2.8 to 7.0%, is higher in women, and changes with age [21, 47, 48]. UCAs are often located in the carotid artery [21, 47, 48]. A meta-analysis of aneurysms shows that the prevalence rate of UCAs (3.2%) in patients without complications is higher among women, which may be attributable to the fact that there are more women aged ≥50 years [49]. The present subjects were 24–77 years old, and the prevalence rate of UCAs of those with no medical history of aneurysmal SAH or aneurysm was 10.0%; this is higher than that found in previous autopsy and imaging studies. In the present study, the prevalence rate of UCAs was slightly higher in women than men (10.2% versus 9.8%), but the difference was not significant; furthermore, there was no association between the prevalence rate of UCAs and age. Many aneurysms were located in the C5-6 segments of the carotid artery, which is consistent with other imaging studies. However, this was not the case with autopsy studies possibly because aneurysmal ruptures are usually located on the AcomA, posterior communicating segment of the carotid artery, or middle cerebral artery. In addition, aneurysms in these areas tend to rupture when they are small. The use of high-sensitivity head CTA also aids the discovery of aneurysms at the bend of the carotid siphon. Clinical observations show aneurysms tend to develop in areas with increased hemodynamic pressure, such as at the tops of arterial branches and where vessels bend [50]. The C5-6 segments are partially located in the siphon segment, thereby resulting in aneurysm formation. Since there were only 30 aneurysms, we did not investigate the relationships between aneurysms and CoW variations.

## 6. Conclusions

In the study, head CTA imaging showed significant correlations between certain variations and gender. The prevalence of incomplete variations in the posterior CoW was statistically higher than that of the anterior CoW. As for the overall CoW morphology, no obvious change was observed with respect to sex or ages, supporting the knowledge that CoW is primarily genetically determined. Population with family history of stroke has a significantly higher percentage of incomplete circles compared to literature (such a comparison may generate statistical deviation), and no obvious correlations were observed between the gross CoW morphology and risk factors, which is a reminder that the CoW may be independent of common risk factors and may be related to ischemic events. The prevalence of intracranial aneurysm is comparatively high in people with family history of stroke, but there is no significant relationship between this high rate of occurrence and age or sex. The present study did not investigate the relationship between aneurysm and other factors (variations of CoW, smoking, drinking and genetic factors, etc.). Further studies in a larger sample size are warranted to confirm or refute our findings as well as studies looking at the association of CoW and gender, age, and aneurysms.

## Figures and Tables

**Table 1:** The basic characteristics of the participants.

Characteristic	Males (*n* = 153)	Females (*n* = 128)	Total (*n* = 281)	*p* value
Age (y; mean ± SD)	50.9 ± 11.5	50.8 ± 9.2	50.9 ± 10.5	0.887
Hypertension, *n* (%)	79 (51.6)	80 (62.5)	159 (56.6)	0.067
Diabetes, *n* (%)	15 (9.8)	10 (7.8)	25 (8.9)	0.559
Hypertriglyceridemia, *n* (%)	30 (19.6)	58 (45.3)	88 (31.3)	<0.001
Low HDL-C, *n* (%)	38 (24.8)	17 (13.3)	55 (19.6)	0.015
Smoking, *n* (%)	120 (78.4)	6 (4.7)	126 (44.8)	<0.001
Alcohol use, *n* (%)	81 (52.9)	5 (3.9)	86 (29.9)	<0.001
BMI (kg/m^2^; mean ± SD)	25.67 ± 3.28	26.21 ± 4.91	25.92 ± 3.73	0.221
Overweight, *n* (%)^*∗*^	91 (59.5)	77 (60.2)	168 (59.8)	0.908
Education (y; mean ± SD)	8 ± 2.88	5 ± 3.94	7 ± 3.60	<0.001
Stroke, *n* (%)	10 (6.5)	9 (7.0)	19 (6.8)	0.869

Data presented as *n* (%) of patients or mean ± SD. *p* value showing differences between males and females. ^*∗*^Overweight indicates that obese individuals were included in the overweight individuals.

**Table 2:** Anatomical variations in the anterior Circle of Willis.

	Participant, *n*	Normal circle, *n* (%)	Prevalence of variants, *n*									Incomplete proportion, *n* (%)
			Incomplete						Complete		Total of variants	
			a_1_	a_2_	a_3_	a_4_	b	c	d_1_	d_2_		
Male	153	93 (60.8)	5	4	1	4	43	1^†^	2	0	60	58 (37.9)
Female	128	63 (49.2)	1	11	2	10	39	0	1	1	65	63 (49.2)
Total	281	156 (55.5)	6	15	3	14	82	1	3	1	125	121 (43.1)
*p* value												0.057

† indicates that the variation includes undesirable variation of right A1 segment.

**Table 3:** Anatomical variations in the posterior Circle of Willis.

	Participants, *n*	Normal circle, *n* (%)	Prevalence of variants, *n*											Incomplete proportion, *n* (%)
			Complete		Incomplete								Total of variants	
			a_1_	a_2_	b_1_	b_2_	c_1_	c_2_	c_3_	c_4_	c_5_	d		
Male	153	29 (19.0)	0	4	14	89	1	7	5	3	1	0	124	120 (78.4)
Female	128	26 (20.3)	7	2	18	58	6	3	5	1	1	1	102	93 (72.7)
Total	281	55 (19.6)	7	6	32	147	7	10	10	4	2	1	226	213 (75.8)
*p* value														0.26

**Table 4:** Age and gender specific prevalence of the entire circle morphology.

	Total, *n*	The entire circle morphology			
		EC, *n* (%)	A-PC, *n* (%)	P-PC, *n* (%)	IC, *n* (%)
Age					
<45	76	11 (14.5)	7 (9.2)	33 (43.4)	25 (32.9)
45–64 y	182	29 (15.9)	12 (6.6)	72 (39.6)	69 (37.9)
≥65 y	23	3 (13.0)	1 (4.3)	12 (52.2)	7 (30.4)
Gender					
Male	153	26 (17.0)	7 (4.6)	69 (45.1)	51 (33.3)
Female	128	17 (13.3)	13 (10.2)	48 (37.5)	50 (39.1)

**Table 5:** Comparison with generally healthy Chinese subjects (control study).

	EC	PC	IC
This study, *n* (%)	31 (14%)	108 (50%)	77 (36%)
Control study, *n* (%)	43 (27%)	89 (56%)	28 (17%)
*p* value	0.003	0.28	<0.001

**Table 6:** The risk factors and the Circle of Willis.

	Hypertension		Smoking		Diabetes		Hypertriglyceridemia	
	Yes (159)	No (122)	Yes (126)	No (155)	Yes (25)	No (256)	Yes (88)	No (193)
EC, *n* (%)	25 (15.7)	18 (14.8)	21 (16.7)	22 (14.2)	3 (12.0)	40 (15.6)	16 (18.2)	27 (14.0)
A-PC, *n* (%)	13 (8.2)	7 (5.7)	7 (5.6)	13 (8.4)	2 (8.0)	18 (7.0)	8 (9.1)	12 (6.2)
P-PC, *n* (%)	57 (35.8)^†^	60 (49.2)^†^	58 (46.0)	59 (38.1)	12 (48.0)	105 (41.0)	31 (35.2)	86 (44.6)
IC, *n* (%)	64 (40.3)	37 (30.3)	40 (31.7)	61 (39.4)	8 (32.0)	93 (36.3)	33 (37.5)	68 (35.2)

^†^ *p* < 0.05, the comparison of 49.2% and 35.8%.

**Table 7:** The prevalence of intracranial aneurysm with 95% CI by age and gender.

Age	Male			Female			Total		
		Aneurysms			Aneurysms			Aneurysms	
	Volunteers, *n*	Participants, *n*	Proportion	Volunteers, *n*	Participants, *n*	Proportion	Volunteers, *n*	Participants, *n*	Proportion
			% (95% CI)			% (95% CI)			% (95% CI)
Total	153	15	9.8 (5.0–14.6)	128	14	10.9 (5.5–16.4)	281	29	10.3 (6.7–13.9)
<45 y	43	6	14.0 (3.2–24.7)	33	3	9.1 (2.0–24.0)	76	9	11.8 (4.4–19.3)
45–64 y	93	8	8.6 (2.8–14.4)	89	11	12.4 (5.4–19.3)	182	19	10.4 (6.0–14.9)
≥65 y	17	1	5.9 (0–29.0)	6	0	— (0–46.0)	23	1	4.3 (0–22.0)
*p* value of trend			0.27			0.76^*∗*^			0.38

^*∗*^ *p* represents the comparison of <45 y group and 45–64 y group.
